# Association between Systemic Immune-Inflammation Index and female breast cancer based on NHANES data (2001–2018): A cross-sectional study

**DOI:** 10.1371/journal.pone.0330571

**Published:** 2025-09-04

**Authors:** Juan Xiong, Deju Zhang, Ying Yuan, Chuntao Quan, Ni Xie

**Affiliations:** 1 Department of Family Medicine, The Second People’s Hospital of Deyang, Deyang, Sichuan, China; 2 Graduate Collaborative Training Base of Shenzhen Second People’s Hospital, Heng Yang, Medical School, University of South China, Hengyang, Hunan, China; 3 BioBank, Shenzhen Second People’s Hospital, The First Affiliated Hospital of Shenzhen University, Shenzhen, China; University of Brasilia: Universidade de Brasilia, BRAZIL

## Abstract

Worldwide cancer statistics have shown that breast cancer dominates female cancer incidence and remains a leading cause of death. The Systemic Immune-Inflammation Index (SII) is a new prognostic indicator of systemic inflammation used to assess systemic immune-inflammatory response levels in the human body. It is associated with the prognosis of various diseases, such as malignant tumors, cardiovascular diseases, and autoimmune diseases. Although SII offers valuable information for diagnosing and predicting the risk of female breast cancer (FBC), the association between SII and FBC has not yet been analyzed. Therefore, the relationship between SII and FBC was investigated in this study. Multivariate logistic regression, model fit assessment using Akaike Information Criterion (AIC) and Bayesian Information Criterion (BIC), and smoothing curve fitting were applied to examine the correlation between SII and FBC using data from the National Health and Nutrition Examination Survey (NHANES) 2001-2018. Then the stability of their association was further examined using subgroup analysis and interaction tests among populations. Results showed a positive correlation between SII and FBC in 17,044 participants with age ≥ 20 years. In the fully adjusted model, every 100-unit increase in SII was accompanied by a 3% increased odds of FBC prevalence [OR = 1.03 (95% CI: 1.01, 1.05)]. Individuals in the highest quartile of SII exhibited 44% increased odds of FBC prevalence than those in the lowest quartile [OR = 1.44 (95% CI: 1.11, 1.88)]. Model fitness assessment using AIC and BIC criteria demonstrated that multivariable-adjusted models exhibited better fit compared to unadjusted models for both continuous and categorical SII specifications. Receiver Operating Characteristic (ROC) curve analysis demonstrated that SII exhibited excellent diagnostic capability for breast cancer, with the area under the ROC curve (AUC) of 0.816 (95% CI: 0.801–0.831), comparable to NLR (AUC = 0.816) and neutrophil counts (AUC = 0.815). In disease-specific performance comparison, SII’s predictive ability for breast cancer (AUC = 0.816) was slightly superior to that for hypertension (AUC = 0.799), with the difference being statistically significant (*P *= 0.0407). Our findings confirmed that SII was a promising biomarker associated with FBC prevalence, and it may provide valuable insights into early screening and personalized treatment strategies.

## Introduction

Breast cancer is the most common malignant tumor and the main cause of cancer-related deaths in women around the world [1]. In 2020, approximately 2.3 million new cases of breast cancer and 685,000 deaths of breast cancer were recorded globally and these two parameters have continued to rise over the past decades [2]. The primary treatment modalities for breast cancer involve surgery, radiotherapy, and systemic treatments such as chemotherapy, endocrine therapy, targeted therapy, or a combination of these approaches [3,4]. Despite decades of advances in breast cancer treatment, the therapeutic outcomes remain far from satisfactory, with many patients still facing poor prognoses and treatment resistance. The current treatment strategies often prove inadequate, particularly for advanced-stage or aggressive subtypes of breast cancer, leading to high mortality rates and treatment failures [5–7]. Thus, there is an urgent need to identify novel, accurate, and cost-effective early detection methods to address both the clinical limitations and economic burden imposed on patients in current breast cancer management.

Biomarkers are biological molecules that serve as indicators for physiological and pathological processes. In cancer diagnostics, these molecular signatures are crucial for disease detection, progression monitoring, and treatment response evaluation [8–10]. Several established serum biomarkers, including Cancer Antigen 15–3 (CA15–3) and Carcinoembryonic Antigen (CEA), have shown clinical significance in breast cancer management [11]. However, these conventional markers exhibit limited sensitivity and specificity for early screening, primarily serving as monitoring tools for disease progression and treatment efficacy. There is growing evidence showing that chronic inflammation may contribute to breast cancer development [12]. Elevated levels of inflammatory markers, such as Tumor Necrosis Factor-α (TNF-α), Interleukin-6 (IL-6), and neutrophils, have been observed in HR-positive breast cancer patients, suggesting their potential role as diagnostic indicators [13]. The systemic immune-inflammation index (SII) is an index incorporating platelet, neutrophil, and lymphocyte counts, and has been applied to estimate the pre-treatment balance between inflammatory factors and immune status in people with cancer [14,15]. Notably, researchers found that SII was associated with cancer progression. In 2024, Pang et al. reported that a low baseline SII was associated with better survival outcomes in HER2-positive metastatic breast cancer patients, manifested as prolonged survival time and decreased malignant progression [16]. Additionally, it was proven to be a promising tool for predicting the prognosis of patients with endometrial cancer [17], colorectal cancer [18], bladder cancer [19], and non-small cell lung cancer [20]. Therefore, there is growing interest in the role of SII in cancer prognosis, and it may outperform the neutrophil-to-lymphocyte ratio/platelet-to-lymphocyte ratio (NLR/PLR) as a predictor of breast cancer [21]. To explore the relationship between SII and breast cancer prevalence in the general population, we utilized large-scale population data from 2001–2018 NHANES. Additionally, we employed both Akaike Information Criterion (AIC) and Bayesian Information Criterion (BIC) as model selection criteria to assess the goodness of fit, while also utilizing Receiver Operating Characteristic (ROC) curve analysis to determine the area under the ROC curve (AUC), sensitivity, specificity, Positive Predictive Value (PPV) and Negative Predictive Value (NPV) of SII as a diagnostic biomarker for breast cancer, thereby providing evidence for its potential clinical application in early breast cancer screening.

## Materials and methods

### Study design

**Fig 1:**
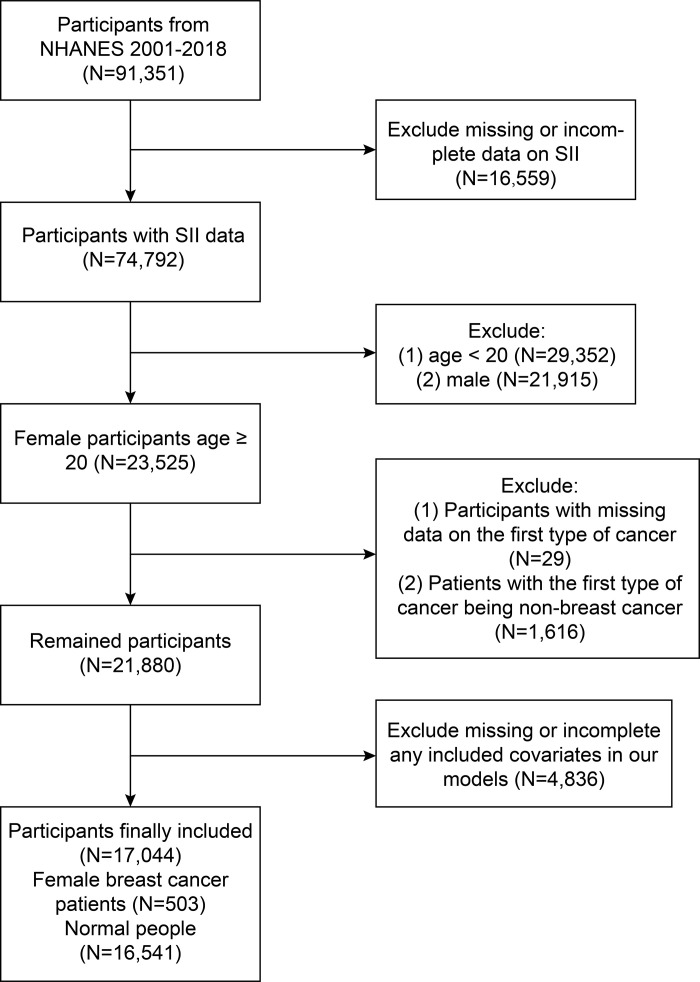
Flow chart of participants selection. NHANES, National Health and Nutrition Examination Survey.

NHANES collects information on the health and nutritional conditions of the noninstitutionalized United States civil population. Our study included data from 91,351 participants during the 9 cycles (2001–2018), among which 74,307 participants were excluded due to missing data, and 17,044 participants (503 breast cancer female participants and 16,541 normal females) were finally involved. Fig 1 shows the full process of the sample exclusion process. All participants gave written informed consent when recruited and the NCHS Research Ethics Review Board approved the study methodology, and no external ethic approval was required for the study to be conducted. The detailed experimental design and data can be obtained from the database (https://www.cdc.gov/nchs/nhanes/; accessed on 12 February 2024).

### Calculation and assessment of SII

The SII is calculated using the following equation: SII = (P * N)/ L, where P, N, and L represent peripheral platelet count, neutrophil count, and lymphocyte count respectively.

### Diagnosis and assessment of breast cancer

In this research, diagnoses of cancer were self-reported through medical questionnaires on medical conditions. All the participants were asked to self-report whether or not they had cancer, based on their visits to a doctor or other health professional. Those who answered “No cancer” were classified as control people, while those answering “YES” were asked to specify their cancer type. Those who answered “breast cancer” were assigned to the breast cancer participants category.

### Study covariates

Based on related studies of current literature [22,23], age, race/ethnicity, educational level, marital status, body mass index (BMI), hypertension status, diabetes status, reproductive health conditions (including age at menarche, pregnancy, and oral contraceptive use), and smoking history were considered major potential confounding factors. Race/ethnicity involves Non-Hispanic White, Non-Hispanic Black, Mexican American, and Other Race. Education level includes below high school, high school, and college or above. Marital status was classified as both sexual partner and asexual partner. BMI measurements were conducted at the mobile examination center. BMI was calculated as weight in kilograms divided by height in meters squared (kg/m^2^). After excluding extreme values, it was categorized into normal weight (< 25 kg/m^2^), overweight (25–30 kg/m^2^), and obesity (≥ 30 kg/m^2^). Hypertension status was classified as having or not having hypertension. Diabetes status was divided into two categories: no and diabetes. Smoking history was classified as never (lifetime <100 cigarettes), former (lifetime ≥ 100 cigarettes but currently quit), and current (lifetime ≥ 100 cigarettes and currently smoking). Finally, the model also included reproductive health status, which included age at menarche (<12 years or ≥ 12 years), ever been in a pregnancy (No or Yes), and oral contraceptive use (No or Yes).

### Statistical analysis

All statistical analyses were conducted using NHANES sampling in accordance with the Centers for Disease Control and Prevention (CDC) guidelines to illustrate the complex multistage survey design. Continuous variables were presented as mean ± standard deviation (SD), whereas categorical variables were expressed as proportions. The chi-squared test was applied to evaluate differences between female breast cancer (FBC) patients and normal females for categorical variables, while the Student’s t-test was used for continuous variables within the covariates. Logistic regression was used to calculate the covariate-adjusted odds ratio (OR) for the association between SII and FBC. After the translation of the SII score from a continuous variable to a categorical variable (quartile), a trend test was conducted to evaluate the trend of linear association between SII and breast cancer. In model I, no covariates were adjusted. Model II was adjusted for age, race/ethnicity, education level, and marital status. Model III was adjusted for the covariates in model II as well as BMI, hypertension status, diabetes status, ever been pregnant, oral contraceptive use, age at menarche, and smoking history. Model fit was assessed using AIC and BIC, where lower values indicate better model fit. ROC curve analysis was performed to evaluate the diagnostic performance of SII, NLR, and neutrophil counts for breast cancer. AUC with 95% CI was calculated to assess discriminative ability. At these thresholds, sensitivity, specificity, accuracy, precision, PPV and NPV were calculated for each marker. We also compared the diagnostic performance of SII between breast cancer and hypertension using DeLong’s test. Subgroup analysis was performed to evaluate the correlations between SII and breast cancer in participants of different ages, race/ethnicity, education level, marital status, BMI, hypertension status, and diabetes status, and interaction tests were employed to explore the consistency of the correlations between subgroups. A smoothing curve fitting clearly presents the relationship between SII and breast cancer. All analyses were performed with R (version 4.2) and Empowerstats (version 2.0) and DecisionLinnc (version 1.0). *P* < 0.05 was considered statistically significant.

## Results

### Baseline characteristics

In this study, a total of 17,044 participants were included, with a mean ± SD age of 48.73 ± 18.09 years, consisting of 2.95% FBC participants. Among them, non-Hispanic white individuals, non-Hispanic black individuals, Mexican Americans, and other ethnicities comprised 43.82%, 19.52%, 17.71%, and 18.95%, respectively. Further, the FBC patients were significantly older than normal individuals (*P* < 0.001), with a majority of Non-Hispanic White (*P* < 0.001) (Table 1). Additionally, Compared to healthy control, breast cancer patients were more likely to have no sexual partners (48.91% vs. 43.36%, *P* = 0.014), ever been pregnant (91.25% vs. 84.96%, *P* < 0.001), without oral contraceptive use (44.93% vs. 33.67%, *P* < 0.001), former smoker (29.42% vs. 18.17%, *P* < 0.001), with a history of hypertension (56.06% vs. 31.82%, *P* < 0.001), and with a history of diabetes (20.87% vs. 10.40%, *P* < 0.001), a lower platelet counts [245.88 ± 62.73 vs. 265.35 ± 68.68, *P* = 0.012], a lower lymphocyte counts [1.88 ± 0.69 vs. 2.19 ± 0.87, *P* < 0.001], and a higher SII value [631.10 ± 447.79 vs. 574.79 ± 359.16, *P* = 0.011]. Nevertheless, no significant differences were observed in education level, age at menarche, BMI, and neutrophil counts.

**Table 1:** Characteristics among female population ≥20 years of age from NHANES 2001–2018 (n = 17044).

Characteristics	All participants (n = 17044)	Without breast cancer (n = 16541)	With breast cancer (n = 503)	*P*-value
**Age at interview, years**	48.73 ± 18.09	48.15 ± 17.93	67.83 ± 11.72	<0.001
**Race/ethnicity, %**				<0.001
Non-Hispanic White	43.82	43.15	65.81	
Non-Hispanic Black	19.52	19.70	13.52	
Mexican American	17.71	18.00	8.35	
Other Race	18.95	19.15	12.33	
**Education level, %**				0.251
Below high school	24.47	24.56	21.47	
High school	22.23	22.23	22.27	
College or above	53.30	53.21	56.26	
**Marital status, %**				0.014
Sexual partner	56.47	56.64	51.09	
Asexual	43.53	43.36	48.91	
**Ever been pregnant, %**				<0.001
No	14.85	15.04	8.75	
Yes	85.15	84.96	91.25	
**Oral contraceptive use, %**				<0.001
No	34.01	33.67	44.93	
Yes	65.99	66.33	55.07	
**Age at menarche, years**				0.694
<12	20.18	20.20	19.48	
≥12	79.82	79.80	80.52	
**BMI, kg/m** ^**2**^	28.00 ± 5.31	28.00 ± 5.31	27.92 ± 5.21	0.732
**Smoking history, %**				<0.001
Never	64.62	64.72	61.23	
Former	18.50	18.17	29.42	
Current	16.88	17.11	9.34	
**Hypertension, %**				<0.001
No	71.69	68.18	43.94	
Yes	28.31	31.82	56.06	
**Diabetes, %**				<0.001
No	89.29	89.60	79.13	
Yes	10.71	10.40	20.87	
**Neutrophil counts,10** ^**3**^ **/µL**	4.33 ± 1.70	4.33 ± 1.77	4.23 ± 1.67	0.181
**Platelet counts, 10** ^**3**^ **/µL**	264.77 ± 68.59	265.35 ± 68.68	245.88 ± 62.73	<0.001
**Lymphocyte counts, 10** ^**3**^ **/µL**	4.33 ± 1.77	2.19 ± 0.87	1.88 ± 0.69	<0.001
**SII**	576.45 ± 362.19	574.79 ± 359.16	631.10 ± 447.79	0.011

Mean ± SD for continuous variables: the *P*-value was calculated by the Student’s t-test; (%) for categorical variables: the *P*-value was calculated by the chi-square test. Abbreviation: SII, systemic immune-inflammatory index; BMI, body mass index.

### Association between SII and FBC

**Fig 2:**
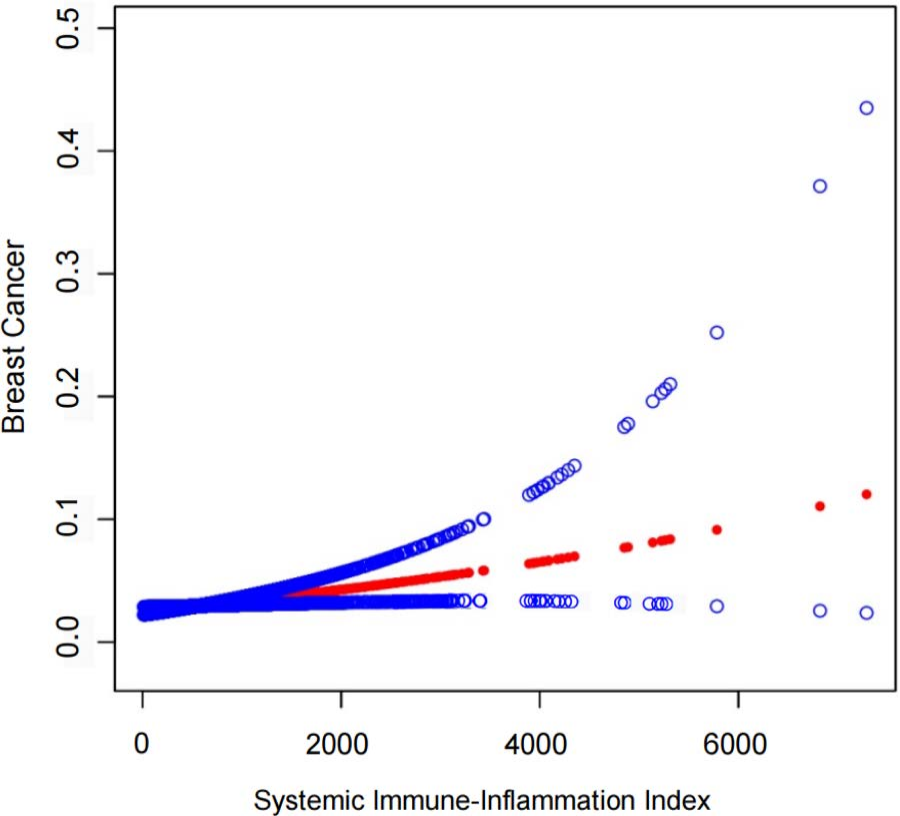
The association between SII and FBC. The solid red line represents the smooth curve fit between variables. Blue bands represent the 95% of confidence interval from the fit. Age, race/ethnicity, education level, marital status, BMI, hypertension status, diabetes status, ever been pregnant, oral contraceptive use, age at menarche, and smoking history were adjusted. Abbreviation: SII = Systemic Immune-Inflammation Index.

As the effect per unit of SII for FBC was small, we examined the linear correlation between SII and breast cancer per 100 units. The results in Table 2 revealed a statistically significant positive association between SII and FBC in the crude model [OR=1.03 (95% CI: 1.01, 1.05)] and partially adjusted model [OR=1.03 (95% CI: 1.01, 1.05)]. After adjusting for all covariates, the FBC prevalence odds rose by 3% for every 100-unit increase in SII [OR=1.03 (95% CI: 1.01, 1.05)]. When the SII was divided into quartiles, their association was still significant (*P* < 0.01). Individuals in the highest quartile of SII showed 44% increased odds of FBC prevalence [OR = 1.44 (95% CI: 1.11, 1.88)] than those in the lowest quartile of SII. Furthermore, a further supported finding was obtained in the smoothed curve fitting (Fig 2). We evaluated model fitness of two SII specifications using AIC and BIC criteria. Results demonstrated improved model fit for multivariable-adjusted models compared to unadjusted models across all SII specifications. Specifically, in the continuous SII/100 analysis, Model I showed AIC and BIC values of 4529.22 and 4544.70 respectively. After adjusting for demographic characteristics (Model II), these values significantly decreased to 3867.60 and 3937.29. Further adjustment for clinical features (Model III) yielded a slight reduction in AIC (3864.77) but a marginal increase in BIC (3996.41), potentially reflecting the trade-off between model complexity and goodness of fit. The categorical SII exhibited similar trends, indicating the robustness of our findings (Table 3).

**Table 2:** Associations between systemic immune-inflammation index and breast cancer.

Outcome	Model I	Model II	Model III
	OR (95% CI, *P*)	OR (95% CI, *P*)	OR (95% CI, *P*)
**Continuous SII/100**	1.03 (1.01, 1.05) P = 0.0006	1.03 (1.01, 1.05) *P *= 0.0085	1.03 (1.01, 1.05) *P *= 0.0094
**Categories**			
Q1(11.23–354.86)	Reference	Reference	Reference
Q2(354.87–498.21)	1.14 (0.88, 1.48), *P *= 0.3194	1.15 (0.88, 1.5.), *P *= 0.3171	1.14 (0.87, 1.49), *P* = 0.3313
Q3(498.27–701.81)	1.08 (0.83, 1.41), *P* = 0.5450	1.09 (0.83, 1.44), *P* = 0.5242	1.10 (0.84, 1.45), *P* = 0.4882
Q4(701.87–7290.94)	1.41 (1.10, 1.81), *P* = 0.0071	1.43 (1.11, 1.87), *P* = 0.0067	1.44 (1.11, 1.88), *P* = 0.0061
***P* for trend**	0.013	0.012	0.010

Model I: no covariates were adjusted. Model II: age, race/ethnicity, education level, and marital status were adjusted. Model III: age, race/ethnicity, education level, marital status, BMI, hypertension status, diabetes status, ever been pregnant, oral contraceptive use, age at menarche, and smoking history were adjusted. Abbreviation: SII, systemic immune-inflammation index, Q means quartile; OR, odds ratio; 95% CI, 95% confidence interval.

**Table 3:** Model comparison using AIC and BIC criteria across different SII specifications.

Specification	Model	AIC	BIC
**Continuous SII/100**	Model I	4529.22	4544.70
	Model II	3867.60	3937.29
	Model III	3864.77	3996.41
**Categories**	Model I	4534.88	4565.85
	Model II	3869.40	3954.57
	Model III	3866.30	4013.43

Lower values of AIC and BIC indicate better model fit while accounting for model complexity. Model I: no covariates were adjusted. Model II: age, race/ethnicity, education level and marital status were adjusted. Model III: age, race/ethnicity, education level, marital status, BMI, hypertension status, diabetes status, ever been pregnant, oral contraceptive use, age at menarche and smoking history were adjusted. Abbreviation: AIC = Akaike Information Criterion; BIC = Bayesian Information Criterion; SII = Systemic Immune-inflammation Index.

### Diagnostic utility and disease specificity of SII

**Fig 3:**
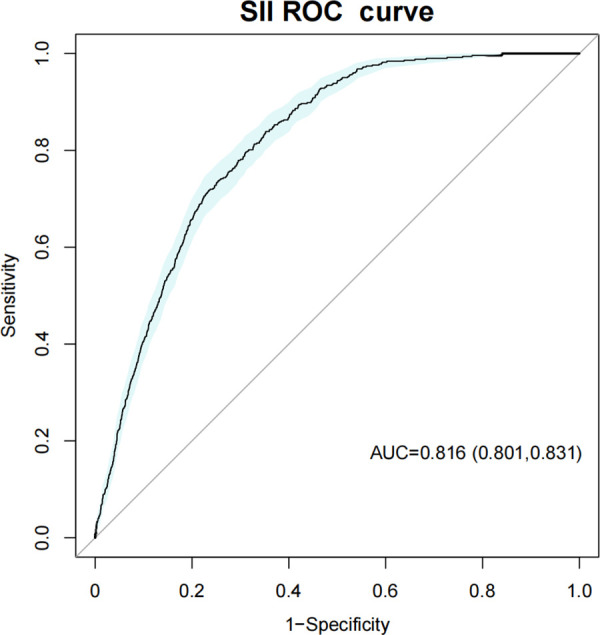
ROC curves evaluating the predictive value of SII for breast cancer. Age and race/ethnicity were adjusted. SII = Systemic Immune-inflammation Index.

To evaluate the diagnostic utility of SII as a biomarker for breast cancer, we compared it with other inflammatory markers (Table 4, S1 Fig, Fig 3). SII, NLR, and neutrophil counts demonstrated comparable discriminative ability with AUC values of 0.816, 0.816, and 0.815, respectively. In the sensitivity-specificity analysis, SII exhibited the highest sensitivity (0.839) with moderate specificity (0.648), neutrophil counts showed the highest specificity (0.774) with lower sensitivity (0.714), and NLR demonstrated intermediate values for both parameters (sensitivity 0.728, specificity 0.761). Regarding predictive values, SII showed the highest negative predictive value (NPV = 0.993) but lowest positive predictive value (PPV = 0.068) among all parameters.

**Table 4:** Performance comparison of three inflammatory markers (SII, NLR, and neutrophil counts) as predictive biomarkers for breast cancer.

Specification	SII	NLR	Neutrophil counts
**AUC (95% CI)**	0.816 (0.801, 0.831)	0.816 (0.801, 0.831)	0.815 (0.800, 0.830)
**Sensitivity**	0.839	0.728	0.714
**Specificity**	0.648	0.761	0.774
**Accuracy**	0.654	0.760	0.772
**Precision**	0.068	0.085	0.088
**NPV**	0.993	0.989	0.989
**PPV**	0.068	0.085	0.088

Age and race/ethnicity were adjusted. AUC = area under the ROC curve, sensitivity represents the ability to correctly identify breast cancer patients, specificity indicates the ability to correctly exclude individuals without breast cancer, precision refers to the proportion of true positives among all samples predicted as positive, accuracy is proportion of correctly classified samples. PPV (positive predictive value) represents the probability that subjects with positive test results truly have the disease. NPV (negative predictive value) represents the probability that subjects with negative test results truly do not have the disease. SII = Systemic Immune-inflammation Index, NLR = Neutrophil to Lymphocyte Ratio.

We also compared SII’s performance between breast cancer and hypertension using ROC curve analysis (Table 5, S2 Fig). SII demonstrated similar overall discriminative ability for both conditions (AUC = 0.816 for breast cancer; AUC = 0.799 for hypertension). For breast cancer, SII showed higher sensitivity (0.839) and lower specificity (0.648) compared to that of hypertension (sensitivity 0.749, specificity 0.724). The predictive values also differed between conditions, with SII for breast cancer showing higher NPV (0.993) but lower PPV (0.068) compared to hypertension (NPV = 0.857, PPV = 0.567).

**Table 5:** Comparison of ROC curve analysis of SII as a predictive biomarker for hypertension and breast cancer.

Specification	Hypertension	Breast cancer
**AUC (95% CI)**	0.799 (0.792, 0.806)	0.816 (0.801, 0.831)
**Sensitivity**	0.749	0.839
**Specificity**	0.724	0.648
**Accuracy**	0.732	0.654
**Precision**	0.567	0.068
**NPV**	0.857	0.993
**PPV**	0.567	0.068

Age and race/ethnicity were adjusted. AUC = area under the ROC curve, sensitivity represents the ability to correctly identify breast cancer patients, specificity indicates the ability to correctly exclude individuals without breast cancer, precision refers to the proportion of true positives among all samples predicted as positive, accuracy is proportion of correctly classified samples. PPV (positive predictive value) represents the probability that subjects with positive test results truly have the disease. NPV (negative predictive value) represents the probability that subjects with negative test results truly do not have the disease. SII’s predictive ability for breast cancer is slightly superior to that for hypertension (*P *= 0.0407). The *P*-value was calculated using DeLong’s test.

### Subgroup analyses

To examine whether the above association was consistent across the general population and to determine any potential variation in population settings, subgroup analyses, and interaction tests were performed and stratified by age, race/ethnicity, education level, marital status, BMI, hypertension, and diabetes (Table 6). Notably, the former exhibited that the correlation between SII and the occurrence of FBC was stable. This association was not significantly affected by age, race/ethnicity, education level, marital status, BMI, hypertension, or diabetes status (all *P* for interaction > 0.05).

**Table 6:** Subgroup analysis of the association between SII/100 and breast cancer.

Subgroup	Breast cancer OR (95% CI, *P*)	*P* for interaction
**Age at interview, years**		0.1903
< 60 years	1.00 (0.95, 1.05), *P *= 0.9767	
≥ 60 years	1.04 (1.01, 1.06), *P* = 0.0011	
**Race/ethnicity, %**		0.4197
Non-Hispanic White	1.02 (0.99, 1.04), *P *= 0.1607	
Non-Hispanic Black	1.05 (0.99, 1.11), *P *= 0.0875	
Mexican American	1.05 (1.01, 1.10), *P *= 0.0241	
Other Race	1.06 (1.00, 1.12), *P *= 0.0433	
**Education level, %**		0.8815
Below high school	1.03 (1.00, 1.07), *P *= 0.0723	
High school	1.03 (1.00, 1.07), *P *= 0.0674	
College or above	1.02 (0.99, 1.05), *P *= 0.1224	
**Marital status, %**		0.2564
Sexual partner	1.02 (0.99, 1.05), *P *= 0.3006	
Asexual	1.04 (1.01, 1.07), *P *= 0.0028	
**BMI, kg/m** ^**2**^		0.4923
<25	1.03 (0.99, 1.06), *P *= 0.1318	
25-30	1.04 (1.01, 1.07), *P *= 0.0057	
≥30	1.01(0.97, 1.05), *P *= 0.5771	
**Hypertension, %**		0.9513
No	1.03 (1.00, 1.06), *P *= 0.0775	
Yes	1.03 (1.00, 1.05), *P *= 0.0202	
**Diabetes, %**		0.3309
No	1.02 (1.00, 1.05), *P* = 0.0354	
Yes	1.05 (1.01, 1.09), *P *= 0.0197	

Age, race/ethnicity, education level, marital status, BMI, hypertension status, diabetes status, ever been pregnant, oral contraceptive use, age at menarche, and smoking history were adjusted. When conducting subgroup analyses, the subgroup factor itself was not adjusted for. Abbreviation: BMI, body mass index; SII, systemic immune-inflammation index; OR, odds ratio; 95% CI, 95% confidence interval.

## Discussion

Our findings indicated that a positive correlation between elevated SII levels and the occurrence of FBC and this association was consistent across different population settings.

This study is highly innovative because it is the first to explore the association between SII and FBC using data from the 2001–2018 NHANES database of US women aged **≥** 20 years. Previous studies have investigated the relationship between preoperative SII and oncological outcomes in patients with FBC using different epidemiological methods and target populations [21,24,25]. A retrospective cohort study of 1489 Chinese patients with primary breast cancer demonstrated that SII may eclipse NLR and PLR as a predictor of breast cancer, especially in the HR-positive subtype [21]. Specifically, we also found that the specificity of the follow-up SII for detecting cancer recurrence was greater than 98.0% at a cut-off of 900. SII-high patients are positively correlated with worse chemotherapy responses and a greater possibility of relapse [21]. Another retrospective research showed that SII predicts prognosis and neoadjuvant chemotherapy efficacy in patients with Human Epidermal Growth Factor Receptor 2 (HER2) positive breast cancer [25]. The complete response group had lower SII than the non-complete response group (587.43 ± 175.97 vs. 821.82 ± 231.58; P = 0.000) [25]. Furthermore, higher preoperative SII was reported as a predictive factor for axillary lymph node metastases in FBC patients [26]. A study with 160 TNBC patients in China suggests that SII may have an independent prognostic significance in triple-negative breast cancer (TNBC) patients. Further investigation is required to identify independent prognostic factors for SII in TNBC, including the use of larger sample sizes, multi-center, and prospective studies [27]. Tang et al. reported that both skeletal muscle index (SMI) and SII were found to independently predict the prognosis of patients with lymph node-positive breast cancer. The combination of SMI and SII may prove to be a more effective prognostic factor than either alone but should be further researched in a larger study [28]. Another meta-analysis involving 2,724 subjects stated that higher SII represented poor overall survival (OS) in cancer patients. Still, the association with disease-free survival/progression-free survival (DFS/PFS) was only significant for breast cancer and ovarian cancer [29].

While SII is indeed a valuable inflammatory indicator, numerous classic markers of inflammation are already in use in clinical practice. A population-based study of 259,435 female participants in the UK Biobank found that markers of inflammation, including C-reactive protein (CRP), monocyte-to-HDL-c ratio (MHR), CRP-to-albumin Ratio (CAR), CRP-to-lymphocyte Ratio (CLR), and neutrophil-to-HDL-c ratio (NHR) were related to FBC [12]. In a study comparing serum levels of inflammatory cytokines between patients with ductal carcinoma and healthy controls, it was found that IL-6 and interleukin-8 (IL-8) content were markedly elevated in the breast cancer group [30,31]. Additionally, TNF-α content was markedly greater in stage III carcinoma patients compared to controls (*P* < 0.01) and was positively correlated to lymph node metastasis (*P* < 0.01) [31]. These findings suggest that these inflammatory cytokines may play a crucial role in the progression and metastasis of breast cancer. Prior research indicated that CRP was generally elevated in breast cancer patients, suggesting its crucial role in the pathophysiology of inflammation in patients with metastatic breast cancer, which is associated with higher mortality [32,33]. A recent retrospective analysis demonstrated that CAR served as an independent prognostic indicator in patients with luminal B subtype breast cancer. The study revealed significantly improved survival outcomes in patients with low CAR levels – both in terms of disease-free survival (DFS) and OS compared to those with high CAR (p < 0.05) [34]. Siersbæk et al. revealed that IL6/STAT3 signaling was decoupled from ER in breast cancer. IL-6 activated STAT3-driven metastasis by co-opting ER-FOXA1-STAT3 enhancers metastasis, suggesting that targeting IL6/STAT3 may have clinical potential in ER+ breast cancer [35]. Kim et al. found that TNF-α promoted tumor progression and invasiveness by regulating tumor remodeling through the induction of matrix metalloproteinas (MMPs), stimulating breast cancer cell activity, and activating JNK and NF-κB pathways [36]. A special subtype of breast cancer was inflammatory breast cancer, which was characterized by negative hormone receptor status and amplification of the HER2 gene. The pathogenesis and progression of IBC largely depended on the tumor microenvironment, featuring an abundance of macrophages, monocytes, and lymphocytes. The tumor and microenvironment cells were well-characterized at the molecular level, demonstrating the major contribution of inflammatory pathways in inflammatory breast cancer tumor biology [37].

In addition to breast cancer, our research findings demonstrated that SII also exhibited significant associations with prostate cancer and non-melanoma skin cancer. In the prostate cancer study, for every 100-unit increase in SII, the prevalence of prostate cancer increased by 2%. Notably, when comparing the Q4 (highest quartile) group with the reference group (Q1), the prevalence of prostate cancer was significantly elevated, with an OR of 1.91 (95% CI: 1.51–2.42, P < 0.001). This association persisted in Model III, with an OR of 1.57 (95% CI: 1.20–2.04, P = 0.0008) (S1 Table). Regarding non-melanoma skin cancer, in Model I without covariate adjustment, SII showed a modest association (continuous SII/100 OR=1.02, 95% CI: 1.00–1.03, P = 0.0105), with the Q4 group demonstrating an increased prevalence relative to the Q1 group [OR=1.46 (95% CI: 1.16–1.84, P = 0.0013)] (S2 Table). These findings demonstrated a correlation that aligned with the growing evidence from hazard ratio (HR) analyses, indicating that SII served as an important prognostic factor across various cancer types. In hepatocellular carcinoma (HCC), the meta-analysis included 10 retrospective studies with 2796 patients, demonstrating that elevated pretreatment SII was significantly associated with poor OS (HR = 1.54, P < 0.001) and earlier time to recurrence (HR = 1.77, *P *< 0.001) [38]. Another study revealed that both SII and alpha-fetoprotein(AFP) were effective predictors of post-interventional recurrence and metastasis, with AUC values of 0.797 and 0.839 respectively, and their combined diagnostic accuracy was notably higher (AUC = 0.910) [39]. Patients with high SII levels demonstrated significantly poorer survival outcomes compared to those with low SII levels. This study inspires us to combine SII with other breast cancer indicators in the next step to improve the predictive accuracy for breast cancer. Across multiple large-scale clinical studies in various cancers, SII proved to be a valuable prognostic marker. Meta-analyses demonstrated that elevated SII was closely associated with poor prognosis, a trend consistently observed across different tumor types. In a meta-analysis of 4,236 gastric cancer patients, high pretreatment SII levels were significantly associated with shortened overall survival (OS) [HR = 1.40 (95% CI: 1.08–1.81)] [40], while in early gastric cancer, SII exhibited excellent predictive capability through ROC curve analysis [AUC = 0.637 (95% CI: 0.551–0.723)] [41]. Similarly, a study of 2,267 non-small cell lung cancer (NSCLC) patients revealed that elevated SII independently predicted poorer OS [HR = 1.52 (95% CI: 1.15–2.00)] [42]. This predictive pattern was also validated in bladder cancer, where analysis of 7,087 patients showed high SII as an independent predictor of worse OS [HR = 1.22 (95% CI: 1.04–1.44)] [19]. These consistent findings across different tumor types emphasized SII’s potential as a universal inflammatory prognostic marker, establishing a solid foundation for its clinical application in multiple cancers. In comparison to traditional inflammatory factors, SII correlates better with inflammatory status preferably and has exhibited more robust prognostic value across multiple types of research [18,43,44]. For instance, Huang et al investigated the predictive accuracy of SII, NLR, NMLR, PLR, and CAR for postoperative survival in colorectal cancer patients. Their analysis showed that SII was one of the most effective and accurate predictors among these markers [21,45]. Our comparative analysis of inflammatory markers revealed that while SII, NLR, and neutrophil counts showed similar overall discriminative ability (as measured by AUC values), they demonstrated distinct sensitivity-specificity profiles. This distinction has important clinical implications. The superior sensitivity of SII compared to both NLR and neutrophil counts positions it as a potentially valuable screening tool for breast cancer. Besides, SII is more suitable for initial screening and exclusion diagnosis of breast cancer compared to other indicators, especially in clinical scenarios where reducing missed diagnoses is critical, as its high sensitivity and high NPV can more effectively capture potential breast cancer patients. The finding aligns with Watson et al. who reported that systemic inflammatory markers with high sensitivity could serve as effective first-line screening tools in cancer detection [46].

In addition to inflammatory markers like SII, recent years have witnessed significant advancements in next-generation sequencing (NGS) technology and long non-coding RNAs (lncRNAs) for breast cancer diagnosis, offering complementary value to existing methods [47]. Circulating lncRNA RP11-445H22.4 has shown specificity (74%) in breast cancer diagnosis. NGS can identify specific genetic alterations driving carcinogenesis, including mutations and gene fusions. NGS provides more detailed molecular information but at higher costs and requires specialized laboratory support [48]. SII, as a cost-effective and simple inflammatory marker, demonstrates sensitivity and comprehensiveness, offering new possibilities for tumor diagnosis. By reflecting the complex interactions between tumors and the immune system, SII provides crucial clinical assessment insights. The research trend points towards further integration with NGS and lncRNA molecular technologies, enabling comprehensive analysis of tumor molecular characteristics, significantly improving diagnostic accuracy and providing precise guidance for personalized treatment decisions.

Recent studies have demonstrated significant interactions between SII and various biomarkers, suggesting complex regulatory networks in systemic inflammation. Wei et al. revealed that SII showed a significant positive correlation with female estradiol levels and was identified by the XGBoost model as one of the key indicators affecting sex hormone levels [49]. Higher levels of estradiol had been associated with increased breast cancer incidence, which explained why elevated SII values indicated higher breast cancer prevalence [50]. Notably, loss of tumor protein p53 (p53) triggered systemic inflammation through promoting WNT ligand secretion and subsequent IL-1β production by tumor-associated macrophages, leading to increased circulating neutrophils. This systemic inflammatory response is reflected by elevated SII values, representing a process driven by genetic alterations in tumor cells (such as p53 loss) [51]. In summary, these findings collectively demonstrated that SII interacted with diverse biological processes from hormonal regulation to cancer progression, providing new insights into the complex relationships between systemic inflammation, endocrine function, and tumor development.

Furthermore, our comparison of SII’s performance between breast cancer and hypertension provides novel insights into its disease-specific characteristics. SII demonstrated high sensitivity and NPV in breast cancer detection. This differential performance across different diseases illustrates the disease-specific characteristics of SII as a diagnostic tool. However, it is essential to emphasize that SII cannot substitute for mammography and breast imaging-reporting and data System (BI-RADS). It may serve as an initial screening tool, where positive findings necessitate further confirmation through mammography and BI-RADS to establish a definitive diagnosis of breast cancer [52]. Implementation of SII as a complementary screening parameter demonstrates optimal utility, particularly in resource-limited settings. From a scientific perspective, the observed association between elevated SII and breast cancer risk provides new insights into the role of systemic inflammation in breast cancer development [53]. From a clinical practice viewpoint, as a diagnostic biomarker, SII exhibits significant methodological advantages, such as minimally invasive acquisition, cost-effectiveness, and accessibility via routine hematological parameters without ionizing radiation exposure [54]. We proposed a tiered clinical implementation framework where baseline SII measurements were incorporated into routine health check-ups, with regular monitoring implemented for individuals with elevated risk. SII could contribute to individualized screening schedules based on specific risk profiles, which could lead to more efficient resource utilization while maintaining screening effectiveness. Future research priorities should focus on prospective validation of these proposed applications, development of standardized protocols, assessment of optimal monitoring frequencies, and evaluation of implementation cost-effectiveness.

Our study has multiple strengths. First, this study has a representative sample and a large sample size. Second, we adjusted for confounding factors, such as socio-demography, reproductive health conditions, diabetes, and blood pressure to produce more reliable results. Finally, this research is innovative due to the lack of exploration of the correlation between SII levels and FBC. However, our findings should be interpreted with caution owing to some limitations. Firstly, the self-reported nature of the NHANES questionnaire limits its ability to provide detailed information on the pathological stages and subtypes of breast cancer among the participants.Secondly, the cross-sectional study design prevents us from building causal relationships. Hence, prospective studies involving more participants are needed to elucidate causality. Thirdly, although some potential covariates were accounted for in our study, the effect of confounding factors still cannot be completely ruled out. Fourthly, we advocate for more detailed subgroup analyses (particularly with stratification by smoking status and alcohol consumption patterns) to examine SII’s role in diverse populations, which would help establish population-specific SII reference ranges and enhance its clinical utility. Lastly, as NHANES participants were exclusively from the United States, the predictive accuracy of our findings may vary when applied to non-U.S. populations due to differences in demographic characteristics, lifestyle factors, and environmental exposures across geographic regions. Future studies should validate these findings in diverse populations across different countries through large-scale prospective cohort studies (n ≥ 10,000) and randomized controlled trials to establish broader external validity and applicability.

## Conclusion

Our study confirmed a positive correlation between elevated SII levels and the occurrence of breast cancer. Nevertheless, further large-scale prospective studies are necessary to confirm the findings as well as establish a causal relationship between SII and FBC.

## Supporting information

S1 Fig ROC curves comparing the predictive performance of different inflammatory markers for breast cancer prevalence. Age and race/ethnicity were adjusted.
(TIF)

S2 Fig ROC curve analysis evaluating the diagnostic performance of predictive models for breast cancer and hypertension. Age and race/ethnicity were adjusted.
(TIF)

S1 Table Associations between systemic immune-inflammation index and prostate cancer. Model I: no covariates were adjusted. Model II: age and race/ethnicity were adjusted. Model III: age, race/ethnicity, education level, marital status, BMI, hypertension status, diabetes status, and smoking history were adjusted. Abbreviation: SII, systemic immune-inflammation index, Q means quartile; OR, odds ratio; 95% CI, 95% confidence interval.
(DOCX)

S2 Table Associations between systemic immune-inflammation index and non-melanoma skin cancer. Model I: no covariates were adjusted. Model II: age and race/ethnicity were adjusted. Model III: age, race/ethnicity, education level, marital status, BMI, hypertension status, diabetes status, and smoking history were adjusted. Abbreviation: SII, systemic immune-inflammation index, Q means quartile; OR, odds ratio; 95% CI, 95% confidence interval.
(DOCX)

S1 File Original Data. Raw experimental data supporting all analyses presented in this study.
(XLSX)
