# Supplementary figures and images for: Association between Systemic Immune-Inflammation Index and female breast cancer based on NHANES data (2001–2018): A cross-sectional study
Source: PLoS One. 2025 Sep 4;20(9):e0330571. doi: 10.1371/journal.pone.0330571 (PMC12410753; doi:10.1371/journal.pone.0330571)

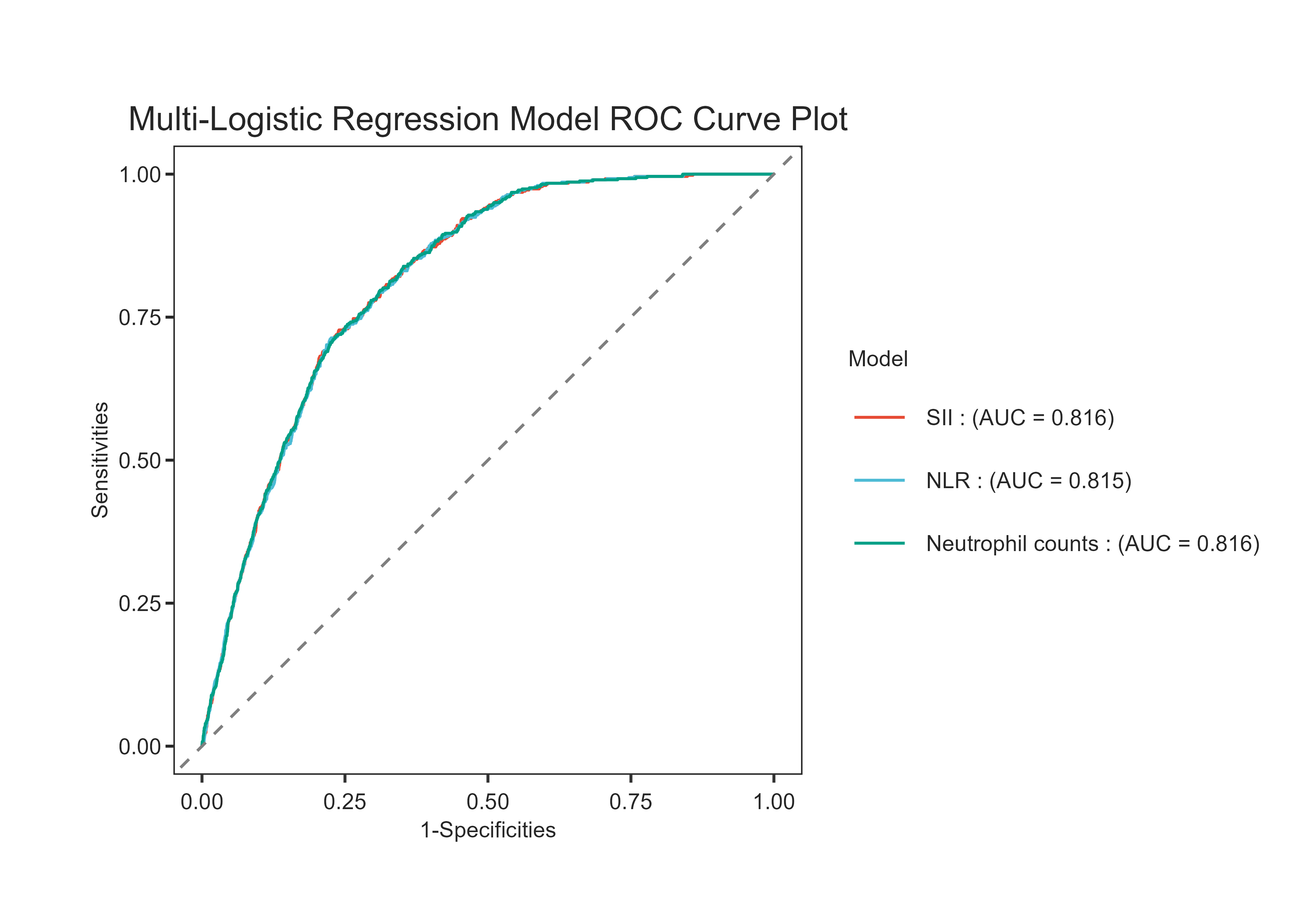

Supplement: S1 Fig — Age and race/ethnicity were adjusted. (TIF) [file pone.0330571.s001.tif]

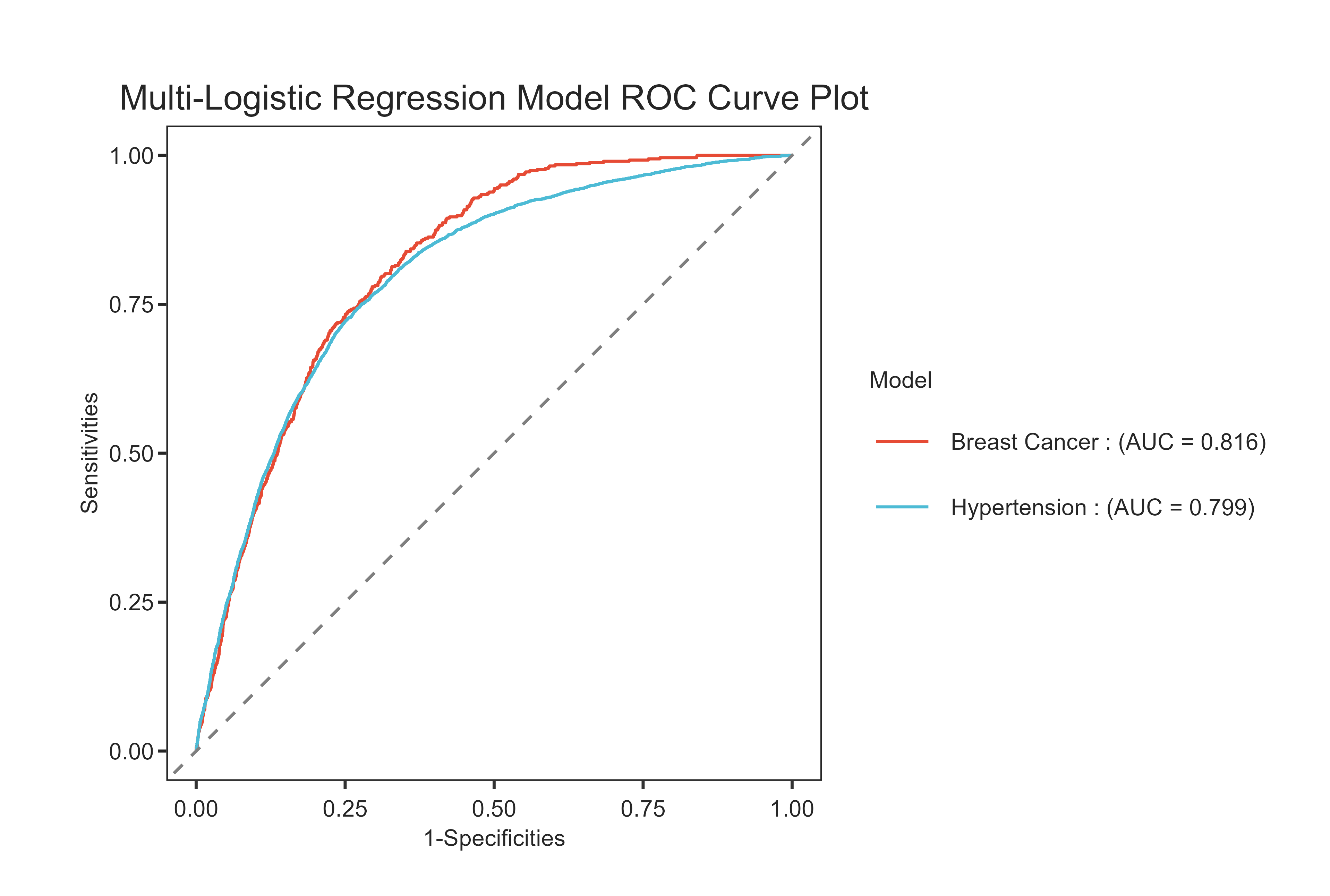

Supplement: S2 Fig — Age and race/ethnicity were adjusted. (TIF) [file pone.0330571.s002.tif]
