# Supplementary material for: Association between Systemic Immune-Inflammation Index and female breast cancer based on NHANES data (2001–2018): A cross-sectional study
Source: PLoS One. 2025 Sep 4;20(9):e0330571. doi: 10.1371/journal.pone.0330571 (PMC12410753; doi:10.1371/journal.pone.0330571)
Supplement: S1 Table — Model I: no covariates were adjusted. Model II: age and race/ethnicity were adjusted. Model III: age, race/ethnicity, education level, marital status, BMI, hypertension status, diabetes status, and smoking history were adjusted. Abbreviation: SII, systemic immune-inflammation index, Q means quartile; OR, odds ratio; 95% CI, 95% confidence interval. (DOCX) [file pone.0330571.s003.docx]

**Table S1:** **Associations between systemic immune-inflammation index and prostate cancer.**

| **Outcome** | **Model I** | **Model II** | **Model III** |
| --- | --- | --- | --- |
|  | **OR (95% CI, *P*)** | **OR (95% CI, *P*)** | **OR (95% CI, *P*)** |
| **Continuous SII/100** | 1.02 (1.01, 1.04) P = 0.0073 | 1.01 (1.00, 1.03) *P* = 0.0760 | 1.01 (1.00, 1.02) *P* = 0.0710 |
| **Categories** |  |  |  |
| Q1 | Reference | Reference | Reference |
| Q2 | 1.09 (0.83, 1.41), *P* = 0.5437 | 1.26 (0.95, 1.67), *P* = 0.1047 | 1.26 (0.95, 1.67), *P*=0.1106 |
| Q3 | 1.24 (0.96, 1.60), *P* = 0.1044 | 1.24 (0.94, 1.64), *P* = 0.1229 | 1.25 (0.94, 1.65), *P*= 0.1192 |
| Q4 | 1.91 (1.51, 2.42), *P* <0.0001 | 1.56 (1.20, 2.02), *P* = 0.0008 | 1.57 (1.20, 2.04), *P*= 0.0008 |
| ***P* for trend** | <0.001 | 0.001 | 0.001 |

Model I: no covariates were adjusted. Model II: age and race/ethnicity were adjusted. Model III: age, race/ethnicity, education level, marital status, BMI, hypertension status, diabetes status, and smoking history were adjusted. Abbreviation: SII, systemic immune-inflammation index, Q means quartile; OR, odds ratio; 95% CI, 95% confidence interval.
