# Supplementary material for: Association between Systemic Immune-Inflammation Index and female breast cancer based on NHANES data (2001–2018): A cross-sectional study
Source: PLoS One. 2025 Sep 4;20(9):e0330571. doi: 10.1371/journal.pone.0330571 (PMC12410753; doi:10.1371/journal.pone.0330571)
Supplement: S2 Table — Model I: no covariates were adjusted. Model II: age and race/ethnicity were adjusted. Model III: age, race/ethnicity, education level, marital status, BMI, hypertension status, diabetes status, and smoking history were adjusted. Abbreviation: SII, systemic immune-inflammation index, Q means quartile; OR, odds ratio; 95% CI, 95% confidence interval. (DOCX) [file pone.0330571.s004.docx]

**Table S2:** **Associations between systemic immune-inflammation index and non-melanoma skin cancer.**

| **Outcome** | **Model I** | **Model II** | **Model III** |
| --- | --- | --- | --- |
|  | **OR (95% CI, *P*)** | **OR (95% CI, *P*)** | **OR (95% CI, *P*)** |
| **Continuous SII/100** | 1.02 (1.00, 1.03), *P* = 0.0105 | 0.99 (0.96, 1.01), *P* = 0.2092 | 0.99 (0.97, 1.01), *P* =0.2667 |
| **Categories** |  |  |  |
| Q1 | Reference | Reference | Reference |
| Q2 | 1.17 (0.92, 1.49), *P* = 0.1982 | 0.96 (0.75, 1.24), *P* = 0.7767 | 0.95 (0.74, 1.2), *P* = 0.6889 |
| Q3 | 1.29 (1.02, 1.63), *P* = 0.0365 | 0.90 (0.70, 1.14), *P* = 0.3745 | 0.90 (0.71, 1.15), *P*= 0.4129 |
| Q4 | 1.46 (1.16, 1.84), *P* = 0.0013 | 0.85 (0.67, 1.08), *P* = 0.1933 | 0.86 (0.67, 1.09), *P*= 0.2118 |
| ***P* for trend** | <0.001 | 0.148 | 0.185 |

Model I: no covariates were adjusted. Model II: age and race/ethnicity were adjusted. Model III: age, race/ethnicity, education level, marital status, BMI, hypertension status, diabetes status, and smoking history were adjusted. Abbreviation: SII, systemic immune-inflammation index, Q means quartile; OR, odds ratio; 95% CI, 95% confidence interval.
